# Quantitative Ultrasound Imaging Differences in Multifidus and Thoracolumbar Fasciae between Athletes with and without Chronic Lumbopelvic Pain: A Case-Control Study

**DOI:** 10.3390/jcm9082647

**Published:** 2020-08-14

**Authors:** Jaime Almazán-Polo, Daniel López-López, Carlos Romero-Morales, David Rodríguez-Sanz, Ricardo Becerro-de-Bengoa-Vallejo, Marta Elena Losa-Iglesias, María Bravo-Aguilar, César Calvo-Lobo

**Affiliations:** 1 Faculty of Sport Sciences, Universidad Europea de Madrid, Villaviciosa de Odón, 28670 Madrid, Spain; jaime.almazan@universidadeuropea.es (J.A.-P.); maria.bravo@universidadeuropea.es (M.B.-A.); 2 Research, Health and Podiatry Group, Department of Health Sciences, Faculty of Nursing and Podiatry, Universidade da Coruña, 15403 Ferrol, Spain; daniellopez@udc.es; 3 Facultad de Enfermería, Fisioterapia y Podología, Universidad Complutense de Madrid, 28040 Madrid, Spain; davidrodriguezsanz@gmail.com (D.R.-S.); ribebeva@ucm.es (R.B.-d.-B.-V.); cescalvo@ucm.es (C.C.-L.); 4 Faculty of Health Sciences, Universidad Rey Juan Carlos, 28922 Alcorcón, Spain; marta.losa@urjc.es

**Keywords:** ultrasonography, lower back pain, athletes, grayscale analysis, quantitative ultrasound imaging, lumbar multifidus, cross-sectional area

## Abstract

New trends in ultrasound imaging are focused on exploration of morphology and muscle quality. The main goal of the study was to evaluate the first-order descriptor and echostructure of lumbar multifidus at the L4 vertebral level in athletes with and without chronic lumbopelvic pain (CLPP). A case-control study was performed in 15 semiprofessional athletes with CLPP and 15 without (healthy athletes). Lumbar multifidus echointensity and echovariation were measured for muscle quality assessment. Echostructure was used to evaluate lumbar multifidus cross-sectional area (CSA) at resting and during muscle contraction, respective differences during both phases (CSA^Dif.^), activation patterns, and thoracolumbar fasciae morphology and thickness. Significant differences with a large effect size were observed in quantitative data from CLPP and healthy athletes for left lumbar multifidus CSA^Dif.^ and thoracolumbar fasciae morphology. Categorical data showed statistically significant differences with a small-to-moderate effect size for lumbar multifidus activation pattern and thoracolumbar fasciae morphology. Athletes with CLPP showed a reduced CSA difference between lumbar multifidus contraction and at resting and higher disorganization of thoracolumbar fasciae morphology compared to healthy athletes. These findings suggest the importance of dynamic exploration of the lumbar region and connective tissue in sports performance and injury prevention.

## 1. Introduction

Lower back pain has been recognized as one of the most common health problems in athletes worldwide [1,2]. Despite the general assumption of the importance of physical activity in lower back pain management and prevention, physical activity also could interact as a risk factor depending on volume of training and intensity loads, frequency and periodization of training, individual biomechanics, sport discipline, and competition schedule [3,4]. Athletes who spend a lot of time in training or competition are frequently exposed to repetitive mechanical stress in combination with high speed shifting displacements and lumbar region extreme positions, which leads to an increased risk of injury [5]. In a systematic review, Trompeter et al. [5] showed that athletes had a lifetime prevalence and a one-year prevalence rate of 61% and 24%, respectively, leading athletes to high cost treatments and further loss of training and competition sessions, thus limiting sports performance and quality of life.

Athletic function is frequently determined by the coordination of the kinetic chain body segments, in which the lumbopelvic region, commonly known as “CORE”, works as a linker between extremities in order to maximize effectiveness and efficiency of movement [6]. The coordinated function of deep trunk muscles, such as the lumbar multifidus (LM), protect the lumbopelvic region and lumbar spine [7,8] in addition to facilitating the increase of stiffness by transmitting tensile-forces through elastic elements such as the thoracolumbar fasciae (TLF) [9,10]. In combination with anterolateral abdominal wall muscles, such as transversus abdomins muscle [11,12,13], the lumbar spine stiffness increases to resist flexion moments to the spine [14], maintain stable closure forces to the sacroiliac joint [15,16,17], and improving moments of forces in extension for the lumbar spine [9] in addition to increasing intra-abdominal pressure [8].

The LM muscle is one of the main muscles responsible for inter-vertebral control, neutral and lordosis positions of the lumbar spine [18,19,20], and force distribution from the lower limb [7,21]. Its multifascicular pattern with vertebrae-to-vertebrae insertions have been proposed as key elements in increasing multidirectional stiffness to the lumbar spine [18]. Otherwise, LM and fat infiltration into the paraespinal trunk muscles have also been strongly associated with the presence of chronic lumbopelvic pain (CLPP) as assessed by magnetic resonance imaging [22,23,24,25].

In a similar manner, the TLF as a potential source of pain in the lumbar region as a consequence of micro-injuries due to overuse mechanism, immobilization, or inflammatory processes, has recently been considered [26,27,28]. Along these lines, some hypotheses consider that lower back pain may be a consequence of fibrosis or densification of the connective tissue layers [29], the increase of connective tissue thickness, and the possible consequent restriction of movement [30,31] in addition to the relationship between architectural disorganization of TLF and pain prevalence [32].

Ultrasound imaging is a conservative, portable, non-invasive tool employed in the morphological and architectural evaluation of LM and morphological assessment of the TLF [33,34,35,36]. Ultrasound imaging has been widely employed for LM cross-sectional area (CSA) assessment at resting [21,37,38,39] and thickness (TH) [18,20,40] measurements during dynamic tasks in addition to the thickness [31,32] and morphology [36] of the TLF. However, LM CSA modifications during muscle contraction have not been analyzed, which could help improve the interpretation of morphological modifications and muscle activation patterns during muscle contraction. Regarding qualitative ultrasound imaging analysis, first-order statistical quantitative ultrasound descriptors have been commonly used to assess muscle quality and tissue texture characterization through muscle echogenicity which establish the mean pixel value echointensity by the analysis of the pixel’s gray-distribution in the histogram of a determined selected region of interest (ROI) [35,37,39,41]. Echointensity has been correlated with muscle strength in middle-aged and elderly people [42]. Also, it has been proposed as a potential biomarker in order to detect intra-muscular content disruption [22] in some degenerative musculoskeletal conditions, such as lateral amyotrophic sclerosis [43,44], muscle sarcopenia [45,46], or Achilles tendinopathy [47,48]. The disruption of normal tissue structure observed in acute muscle damage as a consequence of mechanical stress has been associated with a short-term 24-h increases of muscle inter-stitial edema [49,50], as well as degenerative conditions progressing over time towards fat and collagen infiltration being correlated with an increase of echointensity [44,51,52]. Moreover, the recently used first-order statistical parameter of echovariation has been suggested as potential biomarker of tissue homogeneity and uniformity of the pixel’s appearance across the selection area. Echovariation describes the degree of gray dispersion from the average value [53,54] through the relationship between the standard deviation (SD) and the mean pixel value of the histograms displayed from the ROI. Echovariation aims to improve the interpretation of the muscle echogenicity in combination with echointensity, which only considers the average of pixel’s intensity from the region selected.

Therefore, through the use of ultrasound imaging the study aim was to compare differences in the CSA of the LM at L4 vertebral level of both sides during resting and muscle contraction and TLF differences in morphology and thickness between athletes with and without CLPP. Second, the study aimed to determine differences in echogenicity descriptors expressed as a greater echointensity and a lower echovariation in athletes with CLPP compared to athletes without pain in addition to intra-rater reliability of each of the measurements for morphology and echogenicity assessment.

We hypothesized that athletes in the presence of CLPP may show differences in LM echogenicity and homogeneity (echointensity and echovariation) patterns compared to healthy athletes due to the reported association between CLPP and the appearance of disrupted intra-muscular content observed in terms of connective tissue and fatty infiltration [55]. Additionally, we proposed that athletes in pain might present a reduced LM CSA during muscle contraction in addition to differences during muscle contraction and at resting compared to athletes without CLPP.

## 2. Materials & Methods

### 2.1. Study Design

A case-control study based on the STrengthening the Reporting of OBservational studies in Epidemiology (STROBE) statement was conducted [56] in order to compare the echostructure and the echogenicity of the LM between athletes with and without CLPP. The Helsinki Declaration and human experimentation rules [57] were considered and previously, the Ethics Committee of the Universidade da Coruña approved the research. All participants were informed prior to inclusion in the project by providing a written consent form.

### 2.2. Sample Size Calculation

Previously, a pilot study (*n* = 10) had been conducted to calculate the sample size using the mean differences found between groups (*n* = 5, athletes with CLPP group; *n* = 5, athletes without CLPP group) by the use of the G*Power 3.1.9.2 software (G*Power ©, University of Dusseldorf, Düsseldorf, Germany). The cross-sectional area of the right LM (cm^2^) was acquired by ultrasound imaging (mean ± SD) from CLPP group (6.47 ± 0.88 cm^2^) and control group (7.78 ± 1.28 cm^2^) was selected as the main variable previously evaluated. Moreover, a two-tailed hypothesis with an effect size of 1.19, an α error probability of 0.05, power (1-β error probability) of 0.80, and an allocation ratio (N2/N1) of 1 were employed for sample size calculation. Thus, after selecting a sample size of 26 participants who were separated into two groups of 13 participants with CLPP and athlete-matched controls without CLPP in addition to a 20% possible loss to follow-up consideration, a total sample size of 30 athletes were recruited.

### 2.3. Participants

A total of 30 male athletes were recruited by the convenience sampling method based on selection criteria and who had been posteriorly evaluated at Universidad Europea de Madrid. The sample was divided into a group of 15 cases (CLPP athletes) and 15 controls (matched paired athletes without CLPP) from May 2018 to July 2018. Based on the criteria previously used by Whittaker et al. [58], the inclusion criteria for study participation consisted of several parameters: (1) males with bilateral non-specific CLPP (an evolution ≥6 weeks located from the iliac crest to the popliteal fossa) with a history of at least one episode per year of recurrent pain in the past two years; (2) 18–55 years of age in order to avoid tissue changes resulting from the influence of advanced age; (3) amateur athletes (with at least 2 h training schedule per week in addition to competing or training once per week or training depending on the discipline); and (4) physical activity level calculated by the International Physical Activity Questionnaire (IPAQ) ≥1500 metabolic equivalents of tasks per minute per week (METs/min/week), which allowed us to classify athletes into moderate or vigorous physical activity level [59]. The control group included subjects without CLPP or history of pain during the last year that were matched with cases based on the demographic descriptive data and selection criteria of physical activity level, sport category, and respiratory distress scores by the Nijmegen questionnaire. Moreover, all participants were asked about dominant side, hand-throw side, and foot-jump side in order to homogenize the sample [8].

The exclusion criteria were determined by several parameters: (1) presence of radicular symptoms (such as electrical pain or burning feeling) as tested by the active straight leg raise test [8]; (2) appearance of musculoskeletal or lumbopelvic congenital disorders in the previous year; (3) rheumatism and neuromuscular diseases; (4) body mass index (BMI) ≥30 kg/m^2^; (5) respiratory or neurological pathologies; (6) surgical interventions in lumbar spine and the lower limb; (7) lowerlimb impairments (such as fractures, ankle or knee sprains, chronic ankle instability, lumbar pain associated with a groin, pubic or hip conditions such as inguinal hernia, pubis bone stress or femoroacetabular impignement respectively) [8]; (8) lack of skin integrity or skin compromise in the imaging area; and (9) inability of participants to follow the research instructions [58]. Subjects with a score of £1499 METs/min/week in the IPAQ questionnaire in addition to a score ≥24 points indicating hyperventilatory syndrome as registered by the Nijmegen questionnaire were also excluded from the study [8,58].

### 2.4. Sociodemographic Descriptive Data

Athlete age (years), height (cm), weight (kg), BMI (kg/cm^2^) [60], sport category (basketball, football, or fitness), dominant laterality (right, left), throwing hand-side dominance (right, left), and jumping foot dominance (right, left) were collected as a sociodemographic descriptive data [8]. Additionally, physical activity level, respiratory disturbances, and lumbar disability associated with CLPP were assessed using self-reported questionnaires. First, the International Physical Activity Questionnaire (IPAQ) was applied in order to determine moderate to vigorous physical activity levels through employment of the metabolic equivalents in activities of different intensity per minute per week (METs/min/week) [59,61] in which athletes were categorized as moderate (≥1500 to £2999 METs/min/week) to vigorous (≥3000 METs/min/week). Second, subjects who reported hyperventilatory degree or the presence of hypocapnia with a respiratory distress score obtained by the Nijmegen questionnaire [62] ≥24 points were excluded from the study; it was also used in order to determine the relationship of respiratory issues and LM sonographic features due to the possibility that some respiratory issues may contribute to the dysfunction of people in the presence of CLPP [63]. Last, disability in relation to CLPP was measured by the Roland–Morris Disability Questionnaire (RMDQ) [64], a tool that employs 24 questions in order to assess daily life activity limitation ranging from 0 (no-disability) to 24 (high disability) and having a very good test–retest reliability previously published (Intra-class correlation coefficient (ICC) = 0.874).

### 2.5. Ultrasound Imaging Assessment

Ultrasound imaging examination for image acquisition was executed by an expert physical therapist with more than five years of experience in musculoskeletal ultrasound imaging. Participants were assigned a code prior to completing demographic and questionnaire data in such a way that the evaluator was blinded to the location of the subjects in the groups (athletes with CLPP versus athletes without CLPP).

**Figure 1:**
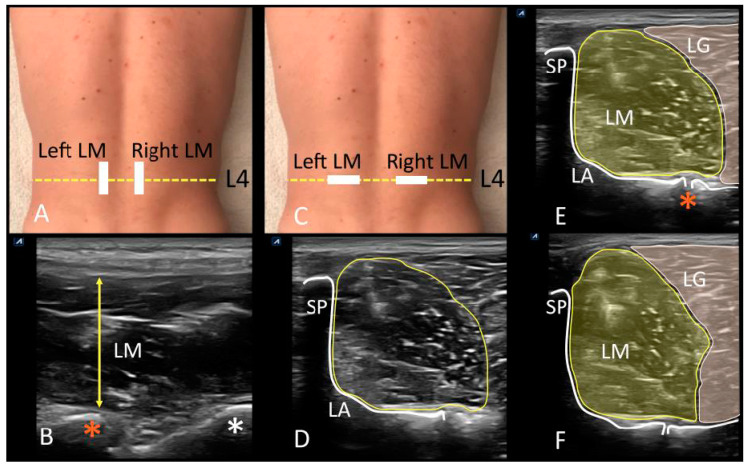
Ultrasonographic evaluation of the lumbar region. (**A**) Probe location for longitudinal assessment of lumbar multifidus at L4 vertebral level (yellow dotted line). (**B**) Longitudinal scanning view of LM (yellow arrow), and L4/5 (orange asterisk) and L5/S1 (white asterisk) facet joints visualization. (**C**) Probe location for transverse scanning of lumbar multifidus at L4 vertebral level (yellow dotted line). (**D**) Transverse scanning view of the lumbar multifidus, and visualization of the L4 spinous process (SP), the laminae (LA) and L4/5 facet joint as measurement landmark, and CSA of LM; (**E**), CSA of lumbar multifidus at resting (LM) and borders delimitation with lumbar longissimus muscle (LG), as well as the white enhancement of the L4 spinous process (SP), the laminae (LA), and facet joint of L4/5 (orange asterisk). (**F**) CSA of lumbar multifidus (LM) during muscle contraction (CAL test) and borders delimitation with lumbar longissimus muscle (LG). Abbreviations: CAL, contralateral arm lift test; CSA, cross-sectional area; LA, laminae; LG, lumbar longissimus muscle; LM, lumbar multifidus; SP, spinous process.

Ultrasound brightness-mode images were acquired using a high quality apparatus (Ecube i7; Alpinion Medical System; Seoul, Korea), which was equipped with a linear probe (Broadband Linear type L3_12T, field of view of 38.4 mm, 128 elements) with a frequency range between 8 and 12.0 MHz and a footprint of 45 mm. Participants rested in a prone position with two pillows placed under the hips to reduce lumbar lordosis keeping the lumbar curvature slightly rectified [18]. The upper limbs were positioned overhead with shoulder abduction of 120° and elbow flexion of 90°, using this position from which LM was assessed at resting and during muscle activation after performing the contralateral arm lift (CAL) test [20]. A pre-fixed preset of 4 cm depth, 10 MHz frequency, 60 point gain, 60 point dynamic range, and 2 foci located at 1.5 and 2.5 cm depths was employed in order to maintain image features constant during image extraction. Maintaining the gain is intended to reduce bias due to gray contrast modification although it negatively affects the delimitation of the fascial edges of the LM, which can be counteracted by recording the video sequence and ultrasound in real time. According to Stokes et al. [18] suggestions, the ultrasound equipment and researcher were situated to the left of the prone athlete, and every exploration started with right side of the spine. The LM muscle was evaluated at L4 lumbar vertebrae, and the iliac crest was used a reference to draw a mark in the spinous process to determine vertebral level. Posteriorly, the probe was placed with longitudinal and cranial orientation in addition to being slightly lateral to the spinous process in order to identify lumbar facet joints. Longitudinal displacements of the probe were carried out from the sacrum to the articular facets of L5/S1 and L4/5, which were used as landmarks (Supplementary Video S1; Figure 1A,B). From this position, transverse scanning of LM was performed with the probe placed transversally (Figure 1C), using the facet joints of L4/5, the vertebral laminae, and the spinous process as landmarks (Video S1; Figure 1D,E). Real-time ultrasonography was used as strategy to identify lateral borders of the LM and lumbar longissimus muscle [18] (Supplementary Video S2; Figure 1E,F). After muscle border delineation, a sequence of video was captured holding the probe firmly with both hands while being careful not to compress the underlying tissue during which time the athletes were informed to perform the CAL task lifting the arm 5 cm from the plinth in series of 3” in order to acquire images from the video of LM at resting (Figure 1E) and during maximum activation (Figure 1F). Finally, gray-scale images were extracted and stored in Digital Imaging and Communications in Medicine (DICOM) format. A reference image of the cross-sectional area (CSA; cm^2^) measured of each LM at resting and contraction due to the advantage of real-time scanning in muscle borders delineation was saved in order not to decrease the reliability of the off-line measurements.

**Figure 2:**
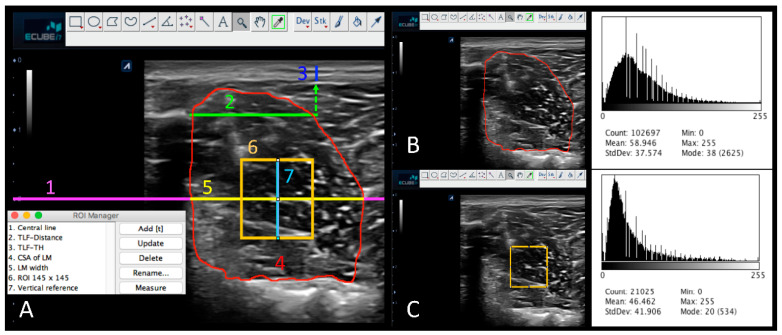
ImageJ measurements of echostructure and first-order descriptors of the lumbar multifidus (LM) and the thoracolumbar fasciae (TLF). (**A**) Representation of the measurement protocol using ImageJ: Central line (1. Central line, magenta), Thoracolumbar fasciae distance reference (2. TLF-Distance, green), thickness (TH) of the thoracolumbar fasciae (3. TLF-TH, blue), cross-sectional area (CSA) of the lumbar multifidus at resting (4. CSA of LM red), width of the lumbar multifidus (5. LM width, yellow), region of interest (ROI) of lumbar multifidus at midpoint (6. ROI 145 × 145 pixels, orange), and vertical line reference for ROI adjustment (7. Vertical reference, cyan). (**B**) CSA of LM histogram displayed. (**C**) ROI of LM histogram displayed.

The version 2.0 of the ImageJ software (United States National Institutes of Health; Bethesda, Maryland, USA) was used to process all data measuring the sonographic of the LM structure and morphology in addition to the echogenicity and tissue homogeneity assessment off-line [8,65] (Figure 2A). An image protocol measurement of 14 steps was designed with the aim of systematizing data extraction (Table 1), in addition to being carried out by a researcher who was blinded to group allocation. Structural measurements of the thickness (TH) in cm and morphology (MPH) of the TLF were analyzed at resting, and the cross-sectional area (CSA, cm^2^) of LM both at resting and at the point of maximum muscle contraction in addition to their differences were obtained. First-order statistical ultrasound parameter included the mean value of pixel reflection or echointensity, and the echovariation of the LM at resting from the CSA and the selected ROI (Figure 2B,C). As recommended by Ríos et al. [53], the echointensity was calculated by the relationship between standard deviation and the echointensity (Echointensity = SD/echointensity *100) in order to obtain information about tissue homogeneity.

**Figure 3:**
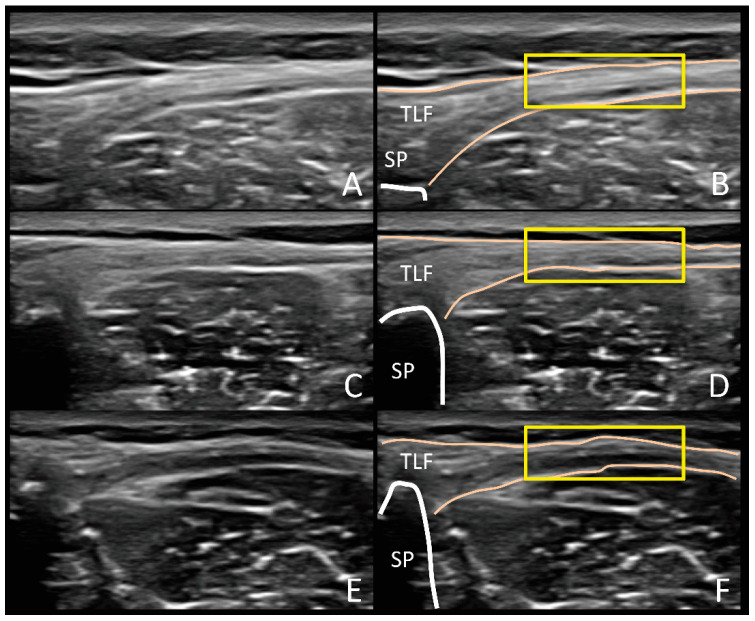
Ultrasound imaging assessment of thoracolumbar fasciae morphology and categorization based on Likert-scale type which considers fascial lines delimitation and organization, echogenicity and the ability to draw a rectangular box around the hyperechoic fascial layers (organized). The figure shows sample homologous images of a “very organized” ((**A**,**B**); Group = 4, Likert-scale type score = 9), “somewhat organized” ((**C**,**D**); Group = 3, Likert-scale type score = 7) and “somewhat disorganized” ((**E**,**F**); Group = 3, Likert-scale type score = 5) thoracolumbar fasciae. Abbreviations: SP, spinous process; TLF, thoracolumbar fasciae.

First, all images were converted from pixels to cm using the reference cm scale from the ultrasound tool, and the ROI manager tool was selected to incorporate every measurement in the software. Second, a transverse line as a central reference at 2 cm deep occupying the entire image was drawn for the subsequent location of the ROI. Based on the method of Bishop et al. [31], a second transverse line 2 cm away from the L4 spinous process in a lateral direction was drawn as a reference point for the measurement of the deep subcutaneous tissue and TLF TH for which the caliper was placed at the most superficial of the deep subcutaneous tissue and the deepest point of the TLF (TLF-TH, cm). Additionally, a 10-point Likert-type scale was used to categorize the morphology of the TLF with 1 corresponding to a “very disorganized” appearance and 10 to a “very organized” pattern, which was defined as the possibility to delineate a rectangular box in the TLF [36] (Figure 3). Posteriorly, scores were organized to classify the morphology (TLF-MPH) into four groups: (1) Group 1: very disorganized, ratings from 1 to 3; (2) Group 2: somewhat disorganized, ratings from 4 to 5; (3) Group 3: somewhat organized, rating from 6 to 7; and (4) Group 4: very organized, rating from 8 to 10 [36]. Third, the CSA of the LM at resting (CSA^Rest^, cm^2^) was delineated, and the subsequent histogram was displayed for echointensity (CSA^EI^) and echovariation (CSA^EV^) variables by using the inner border of the LM regarding longissimus muscle, the subcutaneous fat tissue, and the L4 vertebral laminae in addition to the L4/5 joint facet. Posteriorly, the CSA was incorporated into the ROI manager that was used as a reference for posterior LM width calculations in which the most medial (L4 vertebral laminae contact) and lateral (LM-longissimus border) points of the correspondent central line reference at 2 cm deep with the CSA were determined (LM width). Therefore, an ROI of 145 × 145 pixels (21,025 total pixels) was specified and located at the mid-point of the previous described LM width line of reference. Moreover, to increase specificity of ROI location at the center of LM, a vertical line of 1.13 cm was delineated by forming a cross at the mid-point of the LM width and the ROI box. Subsequently, the histogram was displayed to acquire the mean pixel value (ROI^EI^) and the echovariation (ROI^EV^). Last, the CSA of the LM at CAL was calculated from images acquired during the CAL test (CSA^CAL^, cm^2^) in addition to the difference of the LM at resting and during muscle contraction (CSA^Dif.^ = CSA^CAL^ − CSA^Rest^; cm^2^).

### 2.6. Statistical Analyses

The Statistical Package of Social Sciences (SPSS 24.0v of IBM; Armonk–New York; IBM–Corp, NY, USA) was used for statistical analyses with a α error of 0.05 and a statistically significant *p*-value lower than 0.05 with a confidence interval (CI) of 95%.

Quantitative variables were analyzed using the Shapiro–Wilk test in order to evaluate normality distribution. Parametric data (adjusted to the Shapiro–Wilk test with a *p*-value ≥ 0.05) are presented by the use of mean ± SD and range (minimum–maximum), and differences between groups were analyzed with the Student’s *t*-test for independent samples. Non-parametric data (Shapiro–Wilk test with a *p*-value < 0.05) are described using the median ± interquartile range (IR) and range. In addition, differences between groups were analyzed by Mann–Whitney U test for independent samples.

Categorical data are presented using frequencies (*n*) and percentages (%) to describe data and differences between groups, being posteriorly analyzed using the chi-square test (*χ^2^*) or Fisher exact test for dichotomous variables. The right and left LM-CSA differences were recoded into a different categorical variable named LM activation pattern (ACT.PATT) in which negative values (−) and positive (+) values were tagged as normal and abnormal muscle activation patterns, respectively, and compared by the Fisher exact test. The effect size was determined using Cohen’s d for quantitative data and Cramer’s V for categorical data, and the results were categorized into small (d from 0.20 to 0.49), medium (d from 0.50 to 0.79), and large (d > 0.8) effect sizes [66].

Regarding intra-rater reliability analyses, the intra-class correlation coefficient (ICC) was calculated according to three repeated measurements using a randomized effects model with absolute concordance in order to detail reliability coefficients for each outcome measurement [67]. ICC was interpreted as poor (<0.40), fair (0.40–0.59), good (0.60–0.74), and excellent (0.75–1.00) [68]. Following Portney and Watkins’ recommendations [69], an ICC >0.90 may improve the correct probability for the reliability of outcome measurements. Furthermore, Cronbach’s alpha, standard error of measurements (SEM) and minimum detectable changes (MDC = √2 × 1.96 × SEM) were determined following Bland and Altman’s recommendations [70].

## 3. Results

### 3.1. Homogeneity of the Groups

Thirty subjects were recruited (athletes with CLPP, *n* = 15; athletes without CLPP, *n* = 15) showing a total sample age distribution 26.50 ± 7 (20–35). Athletes reported moderate (9, 30%) and vigorous (21, 70%) physical activity levels. Statistically significant differences were not shown between groups (*p* > 0.05) for quantitative (Table 2) and categorical descriptive data (Table 3).

### 3.2. Intra-Rater Reliability

All outcome measurements showed adequate reliability from good to excellent based on three repeated measurements except for the Right LM Pixels Count-CSA parameter, which revealed a poor reliability according to the Cronbach α, ICC, SEM, and MDC (Table 4).

### 3.3. First-Order Statistical Ultrasound Parameters and Echostructure Ultrasound Imaging Variables

Left LM CSA differences between resting and muscle contraction (CSA^Dif.^ = CSA^CAL^ − CSA^Rest^) showed statistically significant differences (*p* = 0.015) with a large effect size (d = 0.85) for CLLP athletes compared to athletes without CLPP. Additionally, statistically significant differences (*p* < 0.05) with a large effect size (d = 1.43–1.87) were shown for Likert-type scale scores for TLF bilaterally for CLLP athletes with respect to healthy athletes. Categorical dependent variables of ACT.PATT and TLF morphology for both sides also showed significant differences (ACT. PATT, *p* < 0.05 with a small effect size of d = 0.45; TLF-MRPH, *p* < 0.05 with a moderate effect size of d = 0.55–0.73) for athletes with CLPP compared to athletes without CLPP (Table 5). Echotextural outcome measurements did not show any statistically significant differences (Table 6) and the Right LM Pixels Count-CSA parameter was removed (Table 6) according to the poor reliability shown in the intra-rater reliability analysis.

### 3.4. Abnormal Lumbar Multifidus Activation Pattern and Distributions

The Fisher exact test showed statistically significant differences for the recoded categorized variable of activation pattern (ACT.PATT) of the right (*p* < 0.05) and left (*p* < 0.05) lumbar multifidus based on the categorization of the values of the negative (−) and positive (+) values of the differences between muscle contraction and at resting.

## 4. Discussion

In the present case-control study, an analysis of the echostructure and first-order statistical ultrasound descriptors as well as the intra-rater reliability of each measurement was carried out in the lumbar multifidus of athletes with and without CLPP. Echogenicity and homogeneity of ultrasound imaging seemingly did not show differences in first-order descriptors of echointensity and echovariation when they were compared to healthy athletes without CLPP. Nevertheless, athletes with CLPP appeared to show a reduced difference of CSA of LM between resting and muscle contraction during the CAL test, in addition to a higher disorganization of the TLF morphology in comparison with healthy athletes [18,20,54]. Moreover, the thickness of the thoracolumbar fasciae that have been linked with connective tissue fibrosis and pain recurrence in athletes exposed to high-repetitive mechanical loads, did not show differences between athletes with and without CLPP [27,36].

Despite the fact some previous studies have evaluated intra-substance changes in LM in athlete populations with lower back pain [37,39], to the authors’ knowledge, this is the first case-control study focused on the textural analysis of LM using echointensity and echovariation descriptors in addition to analyzing the morphological features of the TLF and LM during resting and contraction [32,35,53]; an intra-rater reliability analysis was carried out in order to improve the reliability of the scores of the measurements. Our results showed an adequate reliability in all measurements of the study except for the Right LM Pixels Count-CSA that was excluded in the analysis of comparisons between groups, in addition to statistically significant differences reported in left LM CSA differences (CSA^Dif.^ = CSA^CAL^ − CSA^Rest^), bilateral significant differences in LM activation pattern (ACT.PATT), TLF Likert-type scale, and TLF morphology in athletes who suffered from bilateral CLPP. Nevertheless, no significant differences were found in the right LM CSA differences (CSA^Dif.^ = CSA^CAL^ − CSA^Rest^) and in the CSA at resting (CSA^Rest^) and during the CAL test (CSA^CAL^) of both sides. Moreover, no statistically significant differences were determined in the outcomes of echotextural measurements in relation to echogenicity and homogeneity of LM intra-muscular content regarding the presence of pain in the athletes with CLPP. In this sense, the results of lumbar disability reported by the group of athletes with CLPP were low, which may explain the similarities observed in first-order descriptors between groups.

### 4.1. Ultrasound Imaging Realiability

The measurement reliability evaluation was carried out for a total of six images, three at resting and three during muscle activation, in order to obtain the mean of three measurements for each variable of echogenicity and morphology of LM and TLF [18]. Our results reported a good-to-excellent reliability values (ICC = 0.725–0.982; 95% CI = 0.493–0.991; Cronbach α = 0.720–0.982), in addition to analysis of the SEM and MDC, which support our results. However, adequate reliability was not observed in the Right LM Pixels Count-CSA that was excluded from the analysis between groups. Considering the differences between groups in the measurements of the study, the means difference between case and control groups did not exceed the SEM (Right LM CSA at resting, SEM = 0.262; Left LM CSA at resting, SEM = 0.235; Right TLF thickness, SEM = 0.01; Left TLF thickness, SEM = 0.01; Right LM ROI echointensity, SEM = 3.470; Left LM ROI echointensity, SEM = 3.053; Left LM ROI echovariation, SEM = 7.238) which could be related to errors in the measurements. The results shown in the study must be considered based on the calculation of the sample size carried out for a case-control study. Sample recruitment adapted to a reliability study should be considered in the future to better analyze the reliability of measurements.

Reliability of intra-rater analyses of CSA in LM during muscle contraction, in addition to the assessment of echogenicity through the echointensity and echovariation, have not been previously established. Nevertheless, previous studies reported values of intra-rater reliability measurements were excellent and showed a high intra-class correlation coefficient (ICC) for lumbar multifidus CSA^Rest^ (ICC = 0.93, 95% CI = 0.88–0.96) at the L4 level in adults ranging in age from 60 to 86 years old [71]. Inter-rater reliability measurements have been described in L5 vertebral level in lumbar multifidus CSA^Rest^ (ICC = 0.92, 95% CI = 0.86–0.96) in older adults; however, no studies were performed at the L4 vertebral level [71]. Regarding the TLF-MPH, an excellent inter-rater reliability was determined (Cronbach’s Alpha = 0.98; SEM = 0.10) in 899 measurements performed in 154 subjects when clinicians with different expertise in musculoskeletal ultrasonography were asked to determine the morphology based on a Likert-type scale used for posterior classification into subgroups [36]. Otherwise, ultrasound imaging measures showed an excellent agreement with magnetic resonance imaging (MRI) in the measurement of three lumbar multifidus CSA^Rest^ images at the L4 vertebral level in a small sample of older adults (*n* = 10) with CLPP (Right LM ICC = 0.99, 95% CI = 0.96–0.99; Left LM ICC = 0.99, 95% CI = 0.97–0.99) and without CLPP (Right LM ICC = 0.95, 95% CI = 0.83–0.99; Left LM ICC = 0.97, 95% CI = 0.91–0.99) [33]. Thus, the average LM-CSA measurements reported in both sides in older adults with CLPP (Right LM expressed as mean ± standard deviation (SD)) = 8.48 ± 1.71, 95% CI = 6.55–9.69; Left LM = 8.21 ± 2.93, 95% CI = 6.12–10.31) and without CLPP (Right LM = 8.84 ± 1.06, 95% CI = 8.07–9.60; Left LM = 8.86 ± 0.92, 95% CI = 8.20–9.52) suggest that quantitative ultrasound imaging could be considered as a reproducible method [33]. However, despite the similar results in our results reported and their agreement with previous studies, further studies are needed in order to support the reliability of the measurements, especially in relation to the reliability of the procedure for the assessment of changes in the CSA during muscle contraction and for the assessment of the echogenicity of the LM.

### 4.2. First-Order Statistical Ultrasound Imaging Descriptors

Our results reported no differences in echointensity and echovariation of LM in athletes with CLPP for ROI and CSA measurements. These findings are in agreement with previous studies in which no association between lower back pain and echointensity in different spots disciplines such as American football or ice-hockey players were found [37,39]. Echointensity of the selected regions has been proposed as a useful descriptor reflecting musculoskeletal quality and alterations due to pathological conditions, such as sarcopenia or myoesteatosis in which changes in fibrous and adipose tissue content and distribution are found. [41,72]. These results may be explained by the fact that our sample consisted of mostly young athletes who reported high physical activity levels without any musculoskeletal disorder, degenerative conditions, or back structural injury [22], which could explain the lower predisposition of the alteration of intra-muscular echogenicity despite the chronic pain condition. Otherwise, echovariation has been proposed as a potential biomarker for detecting muscle homogeneity in which a higher variation of echointensity has been associated with healthy muscle patterns [53,54] but no differences were reported in our results. Furthermore, despite the recommendation of Caresio et al. [41] for using a small dimension of ROI’s for a reliable analysis, our results did not show differences between the two different procedures used to explore echogenic composition of LM or in the pixel counting of the selected CSA (Pixels-Count-CSA). Otherwise, due to the lack of information about the distribution of fat infiltration throughout the muscle in degenerative conditions, the two selections used for the echogenicity analysis covered as much LM muscle tissue as possible in order to decrease the risk of missing degenerative muscle regions.

### 4.3. Echostructural Features of Lumbar Multifidus and Thoracolumbar Fasciae

Statistically significant differences between CAL test and at resting position for left LM (CSA^Dif.^ = CSA^CAL^ − CSA^Rest^) were observed between groups, showing lower values in CLPP group at the L4 level. Although no statistically significant differences between groups were observed bilaterally in CSA during resting and CAL test in prone position or in CSA differences (CSA^Dif.^ = CSA^CAL^ − CSA^Rest^) of the right LM, all of these values were shown lower in CLPP group compared to athletes without CLPP. These results may be due to the fact that the study sample consisted of athletes who did not suffer from current limitations in their sport practice. Similar to the tendency showed in our results, Macdonald et al. [20], Zhang et al. [73], and Dickx et al. [74] mentioned a general reduction on LM thickness from resting to muscle recruitment in prone position, but no previous research evaluated changes of CSA during muscle contraction and participants who were included had unilateral lower back pain. On the contrary, Macdonald et al. [75] also reported an increase in LM activation during the CAL test in standing in LM activation in CLPP group, associating this finding with the theory of maladaptive movement disorders and motor control impairments [20,75]. Thereby, the deep stabilizer muscles from the spine may show an increased expression in neuromuscular activity and thickness in order to balance forces and compensations as a consequence of pain states and movement adaptation. According to LM differences between right and left sides, a possible explanation for this result may be due to the fact that the majority of the athletes recruited were right side dominant (dominant side: 24 (80%); handed throw side: 24 (80%)). Previous studies have revealed the relationship between side dominance, sports discipline, and muscle asymmetry. For instance, Hides et al. [76] reported larger lumbar multifidus ipsilateral on the dominant side in elite cricketers, which may be explained by the interaction between the repetition of the biomechanical sports gesture and the derived muscular adaptations. Likewise, Tsuchikane et al. reported a positive correlation between muscle thickness of LM and abdominal wall muscles of the dominant side with the bat swing speed in baseball players, a determining factor in hitting performance significantly correlated with muscle strength of upper and lower limbs [77]. Despite the results, more research is needed to clarify the behavior of trunk muscle activation during functional tasks [18,20,75] in addition to the relationship between lateral dominance and the biomechanical characteristics of the sport discipline with muscle morphological adaptations and motor control [76,77].

Additionally, bilateral statistically significant differences were observed in Likert-type scale scores of TLF and in the categorical variable of the TLF morphology (TLF-MRPH). De Coninck et al. [36] classified the morphological features of TLF into a different levels of organization–disorganization showing a high degree of agreement of consistency between observers. Our results showed that athletes with CLPP presented a higher level of TLF disorganization when compared with healthy matched-pair subjects. Langevin et al. [30] hypothesized that disorganization of connective tissue layers may play an important role in lower back pain pathophysiology. In this context, the decrease in shear strain forces in the TLF observed in subjects with lower back pain were associated with the appearance of impaired neuromuscular control of dorsal trunk muscles [32]. For instance, the presence of lower back pain has been associated with LM abnormal activation in different muscle parts during functional tests such as prone active straight leg raise (PSLR) or supine active straight leg raise (ASLR) [75]. Otherwise, this abnormality in muscle recruitment has also been proposed as a possible pathway for modifying forces that strain connective tissue, promoting long-term changes related to spatial organization and arrangement of TLF [32], which may explain the differences observed in morphology of TLF which could explain the differences in morphology and not in the thickness of the TLF.

### 4.4. Lumbar Multifidus Activation Pattern

Statistically significant differences were determined bilaterally in the LM activation pattern (ACT.PATT) that were categorized as the negative or positive difference between the muscle contraction during CAL test and the resting of the LM, where the positive distribution between the muscular states were considered the expected value. Regarding the difficulty in obtaining quantitative records in the dynamic ultrasound image, categorization of the observed differences between contraction states aims to improve the interpretation of the changes observed during muscle contraction. In this sense, abnormalities in muscle activation pattern and CSA of the LM have been proposed in subjects with lower back pain or chronic pain states [78]. Calvo-Lobo et al. [8] determined differences in the morphology of the thickness of the diaphragm during the respiratory cycle, which may be compatible with differences in the activation pattern in subjects with non-specific CLPP. Further research is necessary to better understand the role of ultrasound imaging assessment in motor control pattern abnormalities during functional task.

### 4.5. Future Studies and Clinical Implications

Our results suggest that CSA differences between muscle activation and resting may be a novel finding in the interpretation of maladaptive movement disorders and motor control impairments. The clinical finding of asymmetries observed in LM CSA morphology at resting and during muscle contraction in patients with lower back pain may help detect signs related to maladaptive movement disorders and motor control abnormalities in order to design better strategies to define therapeutic objectives and progression criteria through rehabilitation programs based on therapeutic exercise. Besides, assessment of thoracolumbar fascia morphology may be used in future studies as a criterion for the subclassification of lower back pain. Future randomized clinical trials should consider differences between states of muscle contraction after interventions programs of strengthening or motor control of lumbar trunk muscles as potential parameters in order to determine neuromuscular motor control restoration in lower back pain [79]. In addition, future studies may incorporate psychosocial factors in order to compare the relationship in the perpetuation of pain between biomechanical factors, such as tissue degeneration, and psychological factors, such as fear of movement in athletes with chronic non-specific pain. Regarding quantitative ultrasound imaging, future studies of intra-muscular content assessment using first and second order quantitative ultrasound imaging descriptors should consider subclinical conditions of lower back pain, such as motor control impairments with or without degenerative structural alterations of the spine or trunk muscles. Additionally, future studies in female athletes analyzing differences between gender should be considered due to the higher muscle echointensity previously reported in women [41], associated with the higher degree in fibrous and adipose intra-muscular content. Otherwise, technological ultrasound imaging advances in the design of systems aimed at improving the interpretation of functional conditions such as stiffness by means of elastography [80] or microvascular flow [81] may contribute in the improvement in the morphological and functional changes in musculoskeletal conditions.

### 4.6. Limitations

The results of this study should be analyzed considering some limitations. First of all, in order to improve internal reliability, a different method of recruiting participants should be considered. Despite good intra-examiner reliability analyses in past studies, the case-control design type of our present study did not include sufficient individuals for sample size calculations according to a reliability study. This is a major limitation of the present study and future reliability studies are necessary according to our prior study [8]. Second, no previous studies have evaluated CSA changes of LM in transverse plane during muscle contraction; however, all of the images were collected using the same indications described before by Stokes et al. [18] and while holding the probe firmly with both hands in order to decrease artifacts derived from movements of the probe during contraction. Furthermore, randomization of imaging order for the assessment of lumbar was not included, which can be considered as a possible bias that interacts with the differences shown in the change in CSA during muscle contraction. Third, further research is needed aiming to standardize protocols and methods for quantitative ultrasound analysis in trunk muscles and differences during muscle contraction in different functional positions. Non-differences in the echointensity and echovariation between groups could be interpreted as an indicator result of homogeneity in the LM intra-muscular content of athletes, but future studies may be proposed in order to increase reliability. Moreover, most of the echogenicity studies have been developed in the elderly population with tissue changes associated with aging such as sarcopenia, which could explain the low differences observed in young athletes regardless the presence of pain. Last, despite current literature recommendations of selecting ROI’s less than 10% of maximum ROI or 15% than maximum selection [41], two different measurements were collected without showing differences in the results depending on the selection. Future research may consider different sizes extractions of different sizes for better adjustment of selections depending on muscle or structure evaluated.

## 5. Conclusions

Athletes in the presence of CLPP did not seem to show differences in first-order descriptors of echointensity and echovariation when they were compared to healthy athletes without CLPP. Nevertheless, athletes with CLPP seemed to show a reduced difference of CSA of LM between resting and muscle contraction during the CAL test in addition to a higher disorganization of the TLF morphology in comparison to healthy athletes. Further research is necessary in order to analyze the intra- and inter-rater reliability of ultrasound imaging measurements based on echogenicity and homogeneity of LM as well as to determine muscle activation patterns abnormalities by employing the differences between muscle contraction and resting in addition to the relationship among sports biomechanics, side dominance, and sonographic morphological differences during static and muscle contraction.

## Figures and Tables

**Table 1:** Image measurements protocol.

ImageJ ultrasound imaging protocol for image measurements
1. Image calibration from pixels to cm of LM at resting
2. Activating the ROI manager tool for storing selections
3. Delimitation of the transverse reference line at 2 cm deep
4. Delimitation of the 2 cm transverse line from the L4 spinous process
5. TLF-TH measurements
6. Categorization of TLF-MPH into 4 groups based on the Likert-type scale
7. CSA of LM delimitation and measurements at resting
8. LM-CSA histogram display
9. Delimitation of the LM width line at 2 cm deep
10. ROI generation of 145 × 145 pixels
11. Delimitation of the vertical reference line for ROI adjustment
12. ROI placement at mid-point of LM width and vertical reference line
13. Histogram display of the LM-ROI
14. CSA of LM delimitation and measurement during the CAL test
15. CSA difference of LM during resting and muscle contraction (CSA^Dif.^)

Abbreviations: CAL, contralateral arm lift test; CSA, cross-sectional area; CSA^Dif.^, CSA difference of LM during resting a muscle contraction (CSA^Dif.^ = CSA^CAL^ − CSA^Rest^); LM, lumbar multifidus; ROI, region of interest; TLF-MPH, morphology of the thoracolumbar fasciae; TLF-TH, thickness of the thoracolumbar fasciae.

**Table 2:** Quantitative descriptive data for athletes with CLPP, healthy athletes and total sample.

Quantitative Data	Total Sample (*N* = 30)	CLPP Athletes (*n* = 15)	Healthy Athletes (*n* = 15)	*p*-Value
Age (years)	26.50 ± 7	28.00 ± 10	24.00 ± 5	NS ^†^
	(20–35)	(20–35)	(20–35)	
Weight (kg)	76.98 ± 9.40	75.00 ± 10.90	78.95 ± 7.48	NS *
	(53–96)	(53–92)	(65–96)	
Height (m)	1.81 ± 0.08	1.81 ± 0.10	1.81 ± 0.07	NS *
	(1.63–1.98)	(1.63–1.98)	(1.68–1.95)	
BMI (kg/m^2^)	24.03 ± 2.14	23.85 ± 4.50	24.07 ± 1.83	NS ^†^
	(17.31–27.15)	(17.31–25.43)	(22.78–27.15)	
IPAQ (METS/min/week)	3889.50 ± 3252.00	4266.00 ± 2856.00	3198.00 ± 2883.00	NS ^†^
	(1935–19272)	(1935–19272)	(2376–9216)	
RMDQ	3.00 ± 3	3.00 ± 3	-	-
	(0–11)	(0–11)	-	
Nijmegen	6.50 ± 8	6.00 ± 12	7.00 ± 6	NS ^†^
	(1–23)	(2–23)	(1–15)	

Abbreviations: BMI, body mass index; CLPP, chronic lumbo-pelvic pain; IPAQ, International Physical Activity Questionnaire; METs, metabolic equivalent index per week; NS, no significant; RDMQ, Roland–Morris Disability Questionnaire. * Mean ± standard deviation and range (min–max) as well as Student´s *t*-test for independent samples were used according to parametric distributions (Shapiro–Wilk test showing a *p*-value ≥ 0.05). ^†^ Median ± interquartile range and range (min–max) as well as Mann–Whitney U test were applied according to non-parametric distributions (Shapiro–Wilk test showing a *p*-value < 0.05). For all analyses, *p* < 0.05 (for a confidence interval of 95%) was considered as statistically significant.

**Table 3:** Categorical descriptive data for athletes with CLPP, healthy athletes and total sample.

Categorical Data		Total Group (*N* = 30)	CLPP Athletes (*n* = 15)	Healthy Athletes (*n* = 15)	*p*-Value
IPAQ category *	Moderate	9 (30%)	3 (20%)	6 (40%)	NS ^†^
	Vigorous	21 (70%)	12 (80%)	9 (60%)	
Sport category	Basketball	9 (30%)	6 (40%)	3 (20%)	NS ^‡^
	Football	1 (3.3%)	0 (0%)	1 (6.7%)	
	Fitness	20 (66.7%)	9 (60%)	11 (73.3%)	
Dominant side	Right	24 (80%)	12 (80%)	12 (80%)	NS ^‡^
	Left	3 (10%)	1 (6.7%)	2 (13.3%)	
	Both	3 (10%)	3 (13.3%)	1 (6.7%)	
Handed throw side	Right	24 (80%)	13 (86.7%)	11 (73.3%)	NS ^‡^
	Left	4 (13.3%)	1 (6.7%)	3 (20%)	
	Both	2 (6.7%)	1 (6.7%)	1 (6.7%)	
Foot jump side	Right	14 (46.7%)	8 (53.3%)	6 (40%)	NS ^‡^
	Left	15 (50%)	6 (40%)	9 (60%)	
	Both	1 (3.3%)	1 (6.7%)	0 (0%)	

Abbreviations: CLPP, chronic lumbo-pelvic pain; IPAQ, International Physical Activity Questionnaire; METs, metabolic equivalent index per week; NS, no significant. ^†^ Frequency and percentage (%) as well as Fisher exact test were used. ^‡^ Frequency and percentage (%) as well as Chi-squared test (*χ^2^*) were used. * Physical activity levels were divided into moderate (<1500 METs/min/week) or vigorous (≥1500 METs/min/week) according to IPAQ. METS were calculated as total index of metabolic equivalents per minute/week for different physical activity levels [66]. For all analyses, *p* < 0.05 (for a confidence interval of 95%) was considered as statistically significant.

**Table 4:** Intra-rater reliability of the outcome measurements.

Outcome Measurements	Cronbach α	ICC (95% CI)	SEM	MDC
Right LM ROI EI (ROI^EI^)	0.968	0.968 (0.942–0.984)	3.470	9.618
Left LM ROI EI (ROI^EI^)	0.974	0.974 (0.953–0.987)	3.053	8.462
Right LM ROI EV (ROI^EV^)	0.969	0.967 (0.940–0.983)	3.347	9.277
Left LM ROI EV (ROI^EV^)	0.853	0.855 (0.735–0.926)	7.238	20.062
Right LM Pixels Count-CSA	0.268	0.268 (−0.338–0.627)	18,845.066	52,235.860
Left LM Pixels Count-CSA	0.981	0.981 (0.965–0.990)	3608.84	10,003.21
Right LM CSA EI (CSA^EI^)	0.974	0.975 (0.954–0.987)	2.690	7.460
Left LM CSA EI (CSA^EI^)	0.969	0.970 (0.944–0.984)	2.520	6.990
Right LM CSA EV (CSA^EV^)	0.969	0.969 (0.943–0.984)	2.528	7.008
Left LM CSA EV (CSA^EV^)	0.958	0.960 (0.926–0.979)	2.340	6.487
Right LM CSA at rest (CSA^Rest^)	0.968	0.968 (0.941–0.984)	0.262	0.728
Left LM CSA at rest (CSA^Rest^)	0.972	0.972 (0.949–0.986)	0.235	0.653
Right LM CSA during CAL (CSA^CAL^)	0.982	0.982 (0.967–0.991)	0.218	0.606
Left LM CSA during CAL (CSA^CAL^)	0.947	0.947 (0.903–0.973)	0.361	1.001
Right thoracolumbar fasciae (TLF) thickness (TH)	0.861	0.862 (0.748–0.930)	0.015	0.043
Left thoracolumbar fasciae (TLF) thickness (TH)	0.904	0.897 (0.809–0.948)	0.014	0.039
Right thoracolumbar fasciae (TLF) Likert-type Scale	0.968	0.966 (0.938–0.983)	0.279	0.378
Left thoracolumbar fasciae (TLF) Likert-type Scale	0.955	0.957 (0.921–0.978)	0.341	0.946
Right thoracolumbar fasciae morphology (TLF-MRPH)	0.720	0.725 (0.493–0.860)	0.523	1.452
Left thoracolumbar fasciae morphology (TLF-MRPH)	0.859	0.852 (0.729–0.924)	0.262	0.728

Abbreviations: EI, echointensity; EV, echovariation; CSA, cross-sectional area; CSA^EI^, echointensity of the cross-sectional area; CSA^EV^, echovariation of the cross-sectional area; CI, confidence interval (Lower and upper limits of the 95% CI); ICC, intra-class correlation coefficient; LM, lumbar multifidus; MDC, minimum detectable change; ROI^EI^, echointensity of the region of interest; ROI^EV^, echovariation of the region of interest; SEM, standard error of measurement.

**Table 5:** Echostructure data for athletes with CLPP, healthy athletes and total sample.

Outcome Measurements		Total Group (*N* = 30)	CLPP Athletes (*n* = 15)	Healthy Athletes (*n* = 15)	*p*-Value CLPP vs. healthy	Effect Size (d)
**Right LM CSA at rest (CSA^Rest^) (cm^2^)**		7.10 ± 1.44 (4.95–10.08)	7.00 ± 1.31 (5.61–9.64)	7.20 ± 1.59 (4.95–10.08)	0.710 *	NS
Left LM CSA at rest (CSA^Rest^) (cm^2^)		7.36 ± 1.38 (5.07–9.79)	7.34 ± 1.40 (5.35–9.79)	7.39 ± 1.41 (5.07–9.14)	0.920 *	NS
Right LM CSA during CAL (CSA^CAL^) (cm^2^)		7.71 ± 1.60 (5.07–10.96)	7.49 ± 1.31 (5.60–9.97)	7.93 ± 1.88 (5.07–10.96)	0.459 *	NS
Left LM CSA during CAL (CSA^CAL^) (cm^2^)		7.98 ± 1.51 (5.59–11.22)	7.71 ± 1.33 (5.59–9.51)	8.24 ± 1.68 (5.64–11.22)	0.349 *	NS
Right LM CSA difference (CSA^Dif.^ = CSA^CAL^ − CSA^Rest^) (cm^2^)		0.61 ± 0.67 (−0.77 to 2.21)	0.49 ± 0.75 (−0.77 to 1.93)	0.73 ± 0.58 (0.12–2.21)	0.325 *	NS
Left LM CSA difference (CSA^Dif.^ = CSA^CAL^ − CSA^Rest^) (cm^2^)		0.61 ± 0.55 (−0.28 to 2.17)	0.37 ± 0.43 (−0.28 to 1.10)	0.85 ± 0.56 (0.03–2.17)	0.015 *	0.85
Right LM activation pattern (ACT.PATT)	Normal	25 (83.3%)	10 (66.7%)	15 (100%)	**0.042 ^†^**	0.45
	Altered	5 (16.7%)	5 (33.3%)	0 (0%)		
Left LM activation pattern (ACT.PATT)	Normal	25 (83.3%)	10 (66.7%)	15 (100%)	**0.042 ^†^**	0.45
	Altered	5 (16.7%)	5 (33.3%)	0 (0%)		
Right thoracolumbar fasciae (TLF) thickness (TH) (cm)		(0.10–0.24)	0.17 ± 0.04 (0.11–0.24)	0.16 ± 0.04 (0.10–0.23)	0.702 *	NS
Left thoracolumbar fasciae (TLF) thickness (TH) (cm)		0.15 ± 0.04 (0.09–0.26)	0.15 ± 0.04 (0.09–0.26)	0.15 ± 0.04 (0.09–0.23)	0.869 *	NS
Right thoracolumbar fasciae (TLF) Likert-type Scale		7.27 ± 1.60 (4–10)	6.33 ± 1.18 (4–8)	8.20 ± 1.42 (6–10)	0.001 *	1.43
Left thoracolumbar fasciae (TLF) Likert-type Scale		7.07 ± 1.70 (4–10)	5.93 ± 1.28 (4–9)	8.20 ± 1.15 (6–10)	0.001 *	1.87
Right thoracolumbar fasciae morphology (TLF-MRPH)	Somewhat disorganized	4 (13.3%)	4 (26.7%)	0 (0%)	**0.011 ^‡^**	
	Somewhat organized	15 (50%)	9 (60%)	6 (40%)		0.55
	Very organized	11 (36.7%)	2 (13.3%)	9 (60%)		
Left thoracolumbar fasciae morphology (TLF-MRPH)	Somewhat disorganized	7 (23.3%)	7 (46.7%)	0 (0%)	**<0.001 ^‡^**	
	Somewhat organized	11 (36.7%)	7 (46.7%)	4 (26.7%)		0.73
	Very organized	12 (40%)	1 (6.7%)	11 (73.3%)		

Abbreviations: ACT.PATT, activation pattern; CLLP, chronic lumbo-pelvic pain; CSA, cross-sectional area; CSA^CAL^, cross-sectional area during contralateral arm lift task; CSA^Dif^, cross-sectional area differences during contraction and rest; CSA^Rest^, cross-sectional area at resting; NS, no significant; TH; thickness; TLF-MRPH, thoracolumbar fasciae morphology. * Mean ± standard deviation and range (min–max) as well as Student´s *t*-test for independent samples were used according to parametric distributions (Shapiro–Wilk test showing a *p*-value ≥ 0.05). ^†^ Frequency and percentage (%) as well as Fisher exact test were used. ^‡^ Frequency and percentage (%) as well as Chi-squared test (*χ^2^*) were used. Cramer´s V test were used. For all analyses, *p* < 0.05 (for a confidence interval of 95%) was considered as statistically significant (**bold**).

**Table 6:** First-order descriptors data for athletes with CLPP, healthy athletes and total sample.

Outcome Measurements	Total Group (*N* = 30)	CLPP Athletes (*n* = 15)	Healthy Athletes (*n* = 15)	*p*-Value CLPP vs. Healthy
Right LM ROI EI (ROI^EI^)	67.19 ± 19.91 (26.51–112.54)	67.83 ± 23.93 (26.51–112.54)	66.55 ± 15.73 (37.27–89.72)	NS
Left LM ROI EI (ROI^EI^)	63.60 ± 17.70 (34.31–102.63)	64.54 ± 18.75 (34.31–102.63)	62.65 ± 17.17 (34.96–91.60)	NS
Right LM ROI EV (ROI^EV^)	67.98 ± 20.86 (36.96–122.43)	65.20 ± 23.12 (36.96–122.43)	70.76 ± 18.71 (44.49–111.73)	NS
Left LM ROI EV (ROI^EV^)	68.42 ± 16.68 (37.98–109.38)	66.75 ± 14.91 (48.70–101.38)	70.09 ± 18.65 (37.98–109.38)	NS
Right LM CSA EI (CSA^EI^)	66.20 ± 16.31 (36.56–94.43)	67.24 ± 19.24 (36.56–94.43)	65.16 ± 13.38 (36.93–87.29)	NS
Left LM CSA EI (CSA^EI^)	64.51 ± 13.97 (42.15–96.69)	66.37 ± 15.58 (42.15–96.69)	62.64 ± 12.40 (44.41–79.31)	NS
Right LM CSA EV (CSA^EV^)	62.80 ± 13.95 (37.26–96.95)	60.24 ± 16.56 (37.26–96.95)	65.36 ± 10.73 (46.66–84.63)	NS
Left LM CSA EV (CSA^EV^)	63.49 ± 11 (45.25–83.79)	61.05 ± 11.02 (45.25–83.79)	65.95 ± 10.79 (48.93–82.93)	NS
Left LM Pixels Count-CSA	67,493 ± 41,809.75 (17,485.82–137,402)	77,632.85 ± 31,897.47 (46,280.67–137,402)	72,606.28 ± 24,068.05 (46,280.67–137,402.00)	NS

Abbreviations: CLLP, chronic lumbo-pelvic pain; EI, echointensity; EV, echovariation; CSA, cross-sectional area; CSA^EI^, echointensity of the cross-sectional area; CSA^EV^, echovariation of the cross-sectional area; LM, lumbar multifidus; NS, no significant; ROI^EI^, echointensity of the region of interest; ROI^EV^, echovariation of the region of interest. * Mean ± standard deviation and range (min–max) as well as Student´s *t*-test for independent samples were used according to parametric distributions (Shapiro-Wilk test showing a *p*-value ≥ 0.05). † Median ± interquartile range and range (min–max) as well as Mann–Whitney U test were applied according to non-parametric distributions (Shapiro–Wilk test showing a *p*-value < 0.05). For all analyses, *p* < 0.05 (for a confidence interval of 95%) was considered as statistically significant.

## Supplementary Materials

The following are available online at https://www.mdpi.com/2077-0383/9/8/2647/s1, Video S1: Ultrasound imaging assessment for lumbar multifidus muscle location, Video S2: Ultrasound imaging assessment of lumbar multifidus muscle contraction.
