# Supplementary material for: Quantitative Ultrasound Imaging Differences in Multifidus and Thoracolumbar Fasciae between Athletes with and without Chronic Lumbopelvic Pain: A Case-Control Study
Source: J Clin Med. 2020 Aug 14;9(8):2647. doi: 10.3390/jcm9082647 (PMC7464501; doi:10.3390/jcm9082647)
Supplement: Supplementary file 1 [file jcm-09-02647-s001.zip › SUPPLEMENTARY FILE/AME Certificate 74652_Quantitative ultrasound imaging differences of multifidus and thoracolumbar fasciae between athletes with and without chro.pdf]

American Manuscript Editors

English Editing Certificate

This document certifies that the manuscript listed below was edited by the expert staff of American Manuscript Editors, all of whom are native English speakers. Moreover, the document was edited for proper English language, grammar, punctuation, and spelling.

Manuscript Title:

Quantitative ultrasound imaging differences of multifidus and thoracolumbar fasciae between athletes with and without chronic lulumbopelvic pain: A case-control study

Authors:

Jaime Almazán-Polo, Daniel López-López, Carlos Romero-Morales, David Rodríguez-Sanz, Ricardo Becerro-de-Bengoa-Vallejo, Marta Elena Losa-Iglesias, César Calvo-Lobo

Certificate Verification Key:

618-897-091-917-123

Project Number:

74652

This certificate may be verified by emailing info@americanmanuscripteditors.com. Documents receiving this certificate should be prepared for publication. However, please note that the author has the ability to accept or reject our suggestions for changes and can make changes after the editing process is complete, all of which can adversely affect the quality of the text after the editing process.

AMERICAN MANUSCRIPT EDITORS
